# Ultrastructural Evaluation of the Human Oocyte at the Germinal Vesicle Stage during the Application of Assisted Reproductive Technologies

**DOI:** 10.3390/cells11101636

**Published:** 2022-05-13

**Authors:** Maria Grazia Palmerini, Sevastiani Antonouli, Guido Macchiarelli, Sandra Cecconi, Serena Bianchi, Mohammad Ali Khalili, Stefania Annarita Nottola

**Affiliations:** 1 Department of Life, Health and Environmental Sciences, University of L’Aquila, 67100 L’Aquila, Italy; arella_935@hotmail.com (S.A.); gmacchiarelli@univaq.it (G.M.); sandra.cecconi@univaq.it (S.C.); serena.bianchi@univaq.it (S.B.); 2 Department of Reproductive Biology, Yazd Institute for Reproductive Sciences, Shahid Sadoughi University of Medical Sciences, Yazd 97514, Iran; khalili59@hotmail.com; 3 Department of Anatomical, Histological, Forensic Medicine and Orthopedic Science, La Sapienza University, 00161 Rome, Italy; stefania.nottola@uniroma1.it

**Keywords:** oocyte, germinal vesicle, human, electron microscopy, assisted reproductive technologies (ARTs)

## Abstract

After its discovery in 1825 by the physiologist J.E. Purkinje, the human germinal vesicle (GV) attracted the interest of scientists. Discarded after laparotomy or laparoscopic ovum pick up from the pool of retrieved mature oocytes, the leftover GV was mainly used for research purposes. After the discovery of Assisted Reproductive Technologies (ARTs) such as *in vitro* maturation (IVM), *in vitro* fertilization and embryo transfer (IVF-ET) and intracytoplasmic sperm injection (ICSI), its developing potential was explored, and recognized as an important source of germ cells, especially in the case of scarce availability of mature oocytes for pathological/clinical conditions or in the case of previous recurrent implantation failure. We here review the ultrastructural data available on GV-stage human oocytes and their application to ARTs.

## 1. Introduction

### 1.1. From the Ovum to the Cicatricula

Questions about the beginning of life have been raised by philosophers and scientists since the fourth century BC with Aristotle, who described embryonic development inside a chicken egg [1]. However, the discovery of the oocytes started in the 19th century: in 1651, the physician William Harvey employed the Latin word “*ovum*” to refer to the beginning of animal life [2], and with his study he introduced the theory “*omnia ab ovo*”, i.e., all animals are produced from ova. However, when he dissected a red deer during the rut, he could not find any visible evidence in the female “*testes*”, so-called at that time for the production of semen [3]. For three centuries (17th–19th), other researchers observed the reproductive organs in mammals following the “egg theory”, trying to identify the ovum and the place of its generation [4]. Among them, the Dutch physician and anatomist Regner de Graaf (1672) mistakenly believed that the egg consisted of the ovarian follicle itself [5], but correctly discovered that a “seminal vapour” was responsible for fertilization after having reached the eggs. The Italian priest, biologist and physiologist Lazzaro Spallanzani further contrasted the theory of spontaneous generation (1765) [6] by demonstrating the importance of the contact between the sperm and the egg. However, he believed in the theory of preformed organs. The egg description was provided in 1824 by the Swiss physiologist Jean-Luis Prevost and the French chemist Jean-Baptiste Dumas, who wrote that, as long as the egg is in the ovary, it contains a brown zone within which a circular yellow spot can be seen [7]. This again surrounds a smaller circular outline, to which Prévost and Dumas gave the name, which it still bears, of “cicatricula”. By putting a frog fertilized egg into the water they could follow the first phases of segmentation, describing a “granulation” ending in the formation of a structure similar to a “raspberry”, including the presence of “black spots”, probably the cell nuclei [8].

### 1.2. The Identification of the Germinal Vesicle

The germinal vesicle was identified in 1825 by the Bohemian physiologist Jan Evangelista Purkinje, who, a few years later, published at the University of Breslaw a treaty where he described the presence and consistency of a vesicular structure found in the hen’s egg [9]. Within each oocyte or “germ”, Purkinje saw a transparent, liquid-filled sphere, which he named the “*vesicula germinativa*” (germinal vesicle), being considered the germ cell. Two years later, the Prussian-Estonian embryologist Ernst Karl von Baer in Leipzig identified the egg cells in mammals [10]. Moreover, he described the process of ovulation and the initial stages of embryo development in the dog. Baer closely examined follicles under a microscope and saw that each one had a small “yellowish-white point” inside. Microscopic examination of the structure observed enabled him to discover that the ovum was hidden within the “*discus proligerus*”, now known as the cumulus oophorus. He removed one of the yellowish structures with the point of a knife and looked at it again under the microscope, revealing the oocyte. The idea that tissues were made up of cells was not yet known, and he called it the “*ovulum*” or “little egg” [11]. The cellular theory by Matthias Jacob Schleiden and Theodor Schwann in 1839 complemented von Baer’s discovery of the mammalian ovum and endorsed the idea that all reproductive processes pass through a cell or comparable, fundamental organic unit [12]. Theodor Schwann argued that the ovum described by von Bear was probably a cell, and the structure named “*vesicula germinativa*” by Purkinje was the cell nucleus, as also confirmed by Vladislav Kruta (1956) [13]. In 1834, Adolph Bernhardt, a student and pupil of Purkinje and later one of the founding fathers of modern histology, observed in the mammalian ovum an analogous structure to the germinal vesicle, finally identifying the cell nucleus [14,15].

In 1835, Rudolph Wagner, while studying the Graafian follicle of the sheep, discovered the presence of a “spot” within the germinal vesicle, which he called “*macula germinativa*” (germinal spot). Wagner assumed that this “spot” was the origin or first stage in the development of the germinal vesicle. In 1839, Gabriel Gustav Valentin, Purkinje’s close collaborator, was the one who confirmed and introduced the term nucleolus for the *macula germinativa*, according to Purkinje. Later, Valentin, based on Purkinje’s and Wagner’s descriptions, conducted his observations and referred to the nucleolus as a “rounded, transparent secondary nucleus” [14].

The long and conflictual history, as described in the 19th century, of the fully grown GV-stage oocyte, discovered a unique cell owning to its size (nearly 0.1 mm or 100 μm diameter), enclosed by a clear “glass-like shell” or zona pellucida provided with a large nucleus—or germinal vesicle—and containing a prominent spherical nucleolus [16].

### 1.3. GV-Stage Oocytes as a Potential Resource in ARTs

ARTs are now routinely applied for female fertility, also connected to delayed childbearing in western countries, and oncofertility problems. The use of GV-stage oocytes gains an option when the availability of mature oocytes is reduced. However, contrasting data about the right approach for using controlled ovarian stimulation (COS) protocols, IVM conditions and cryopreservation strategies limit the potentiality of this approach.

Data from morphological studies may be helpful to untangle the knot. A morpho-functional approach by confocal microscopy detected the presence of double-strand DNA breaks and the diverse activation of a DNA repair response in human GV-stage oocytes from COS or IVM cycles [17]. However, when immature oocytes collected from stimulated ovaries were subjected to vitrification, morpho-functional data evidenced that they survived the vitrification process and retained their potential to mature to the MII stage [18]. It should be noted that alterations to mitochondria redistribution, CG migration, global DNA methylation expression and, especially, spindle and chromosome organization were reported [18]. By contrast, a recent study of the metaphase plate by confocal microscopy revealed that immature human oocytes subjected first to vitrification and then to *in vitro* maturation up to the MII-stage showed a higher percentage of normal metaphase spindle configuration if compared to those matured *in vitro* first and then vitrified [19]. These discrepancies may be solved with the aid of electron microscopy (EM), the gold standard in revealing the ultrastructural characteristics of immature oocytes.

In this review, we aimed to revise available literature on immature oocytes, putting a particular emphasis on (1) the application of electron microscopy (EM) as an essential tool in ultrastructural studies to optimize and validate ARTs in humans and (2) the use of GV-stage oocytes as a useful source of germ cells in ARTs.

## 2. Materials and Methods

### Inclusion and Exclusion Criteria for the Research in MEDLINE–PubMed, Scopus and ISI Web of Science Databases

All original articles were searched from international databases, including PubMed, Scopus and Web of Science. We searched these engines without language or time limitations. The search was performed using eight keywords in English, including “Immature oocytes, Germinal vesicle (GV), morphology, ultrastructure, electron microscopy, Assisted Reproductive Technology (ART), human”. For the ultrastructural description of subcellular structures, the search was enriched by the following terms: nucleus, nuclear membrane, nucleolus, heterochromatin and euchromatin, cortical granules, endoplasmic reticulum, Golgi apparatus, mitochondria, vacuoles, ooplasm, microvilli, perivitelline space, zona pellucida. In this study, the inclusion criteria were: (1) morphological study; (2) the status of oocyte immaturity; (3) the use of immature oocytes for ARTs.

## 3. Ultrastructure of GV-Stage Oocytes

EM observations on human GV-stage oocytes are important for characterizing their ultrastructure, making an essential contribution in highlighting effects connected to patients’ pathologies, or the detrimental action connected to ARTs as COS or cryopreservation. EM is, therefore, useful in the optimization and validation of ART protocols. Focusing on the application of EM on immature human oocytes from different ARTs we here review data available in the literature:

### 3.1. Nucleus

**Figure 1:**
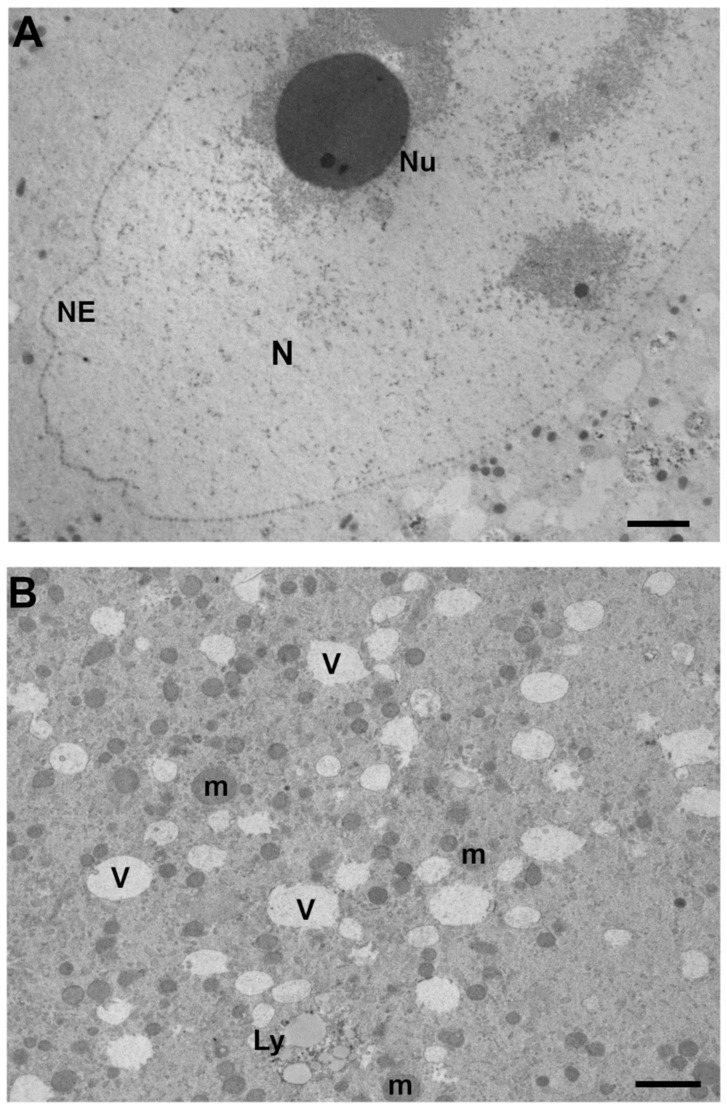
Representative unpublished archived TEM micrographs of GV-stage human oocytes from stimulated ovaries [22,29,30] showing in (**A**) the nucleus (N) delimited by a continuous nuclear envelope (NE) made by a double layer interrupted by electron pale nuclear pores. The nucleoplasm is characterized by a round and electron-dense nucleolus (Nu), dark fibrous clusters of heterochromatin and dispersed euchromatin. TEM, bar: 1 µm; (**B**) the ooplasm rich in electron-lucent vacuoles and small vesicles, delimited by a continuous membrane and sometimes presenting visible inclusions or debris. Numerous round-to-ovoid mitochondria (m) with slightly electron-dense cristae and big secondary lysosome (Ly) with numerous vesicles of different electron-density. TEM, bar: 1 µm.

In immature oocytes aspirated from stimulated ovaries, the germinal vesicle, which was usually spherical and with a diameter of about 20 µm, was located centrally or, in a few oocytes, close to the cell membrane [20]. Similarly, in oocytes collected from patients enrolled in ICSI treatment cycles after COS, the nucleus normally occupied 1.2% of cell volume with a central position in the inner cytoplasm [21]. The nuclear position was, differently, found to be eccentrical in several studies, probably due to an early onset of meiosis resumption after harvesting or COS protocol timing. An eccentrical position of the nucleus in GV-stage human oocytes was described by LM and TEM in both fresh and vitrified/warmed oocytes collected from our group, after a long protocol [22] or by others in IVM cycles from stimulated patients [23,24,25,26]. In all cases, the nuclear membrane was continuous, well visible, two-layered and characterized by the presence of several nuclear pores. The nuclear envelope shape was roundish or folded and enclosed a nucleoplasm with a uniform and pale aspect of finely dispersed euchromatin fibrils, except for the presence of one or more large and electron-dense nucleoli and clusters of heterochromatin. The latter was connected to the nucleoli or organized in patches, sometimes located under the nuclear membrane [20,22,27,28]. Round and highly electron-dense nucleoli were composed almost entirely of fibrillar material; smaller nucleoli were evident in addition to the most evident nucleolus [25]. Dense spherical bodies could be present within their matrices [25]. An unpublished micrograph of the nuclear ultrastructure from a representative fully stimulated human leftover GV-stage oocytes is shown in Figure 1A.

### 3.2. Ooplasm

#### 3.2.1. Mitochondria

GV-stage human oocytes recovered from ovaries from unstimulated patients for further IVM showed that most of the mitochondria were spherical and had sparse cristae [23]. Following ovarian stimulation, they were evenly distributed, although clusters were occasionally seen peripherally [20]. When GV-stage oocytes were collected from PCOS (polycystic ovarian syndrome) patients (no data available about the COS protocol, if any), mitochondria—fewer than at metaphase I—appeared small in size, mostly round but sometimes tubular [31]. In immature oocytes from COS cycles with GnRH (Gonadotropin-Releasing Hormones) agonists and antagonists, most mitochondria localized centrally in the cytoplasm of oocytes with a cluster-like structure; no significant amount of mitochondria was found to localize in their subcortical areas. In fact, the area ratio of “*mitochondria in cytoplasm at peripheral region*” was 0.98%, while that of “*mitochondria in cytoplasm at perinuclear region*” was 7.71% [32]. A similar perinuclear distribution of individual mitochondria and small mitochondrial clusters was found in human leftover GV-stage oocytes from young donors enrolled in an egg donation program [26]. A representative micrograph from our group of mitochondria from a fully stimulated GV-stage human oocyte is shown in Figure 1B.

#### 3.2.2. Mitochondria-Smooth Endoplasmic Reticulum (M-SER) Aggregates

There was a close association between the mitochondria and the smooth endoplasmic reticulum (SER), which appeared as small irregular profiles or sometimes as flattened sacs with a size of about 0.5 to 1 µm [20]. We previously noted the presence of underdeveloped M-SER aggregates in human oocytes after full stimulation [22], while others noted the gradual affiliation of the mitochondria to a smooth membrane of variable ER elements only in MI-stage oocytes [26].

#### 3.2.3. Golgi Apparatus and Smooth Endoplasmic Reticulum (ER)

The Golgi apparatus consisted of aggregates of tubuli and vesicles in human immature oocytes after retrieval from unstimulated patients [23]. This was confirmed by further studies from the group of Sathananthan on immature GV oocytes collected from large antral follicles at laparoscopy from stimulated patients, who described Golgi complexes as the most prominent organelles in the oocytes during the early phases of maturation. They were numerous, hypertrophied and appeared predominantly in the cortical ooplasm, where they were associated with cortical granules [25]. More recently, distinct Golgi apparatus, composed of an interconnected system of cisternae and vesicles, was regularly found in the cytoplasm of leftover human GV oocytes [26]. Interestingly, the cis-trans polarity was not easily distinguishable [26]. Extensive endoplasmic reticulum was visible in human GV oocytes [28], with the prevalence of vesicular smooth ER, as seen in PCOS patients [31].

#### 3.2.4. Cortical Granules (CGs)

The cytoplasm of immature human oocytes collected from ovarian-stimulated women undergoing ARTs for tubal factor infertility contained membrane-bound CGs, with a diameter of 0.3 to 0.5 µm, in varying numbers located close to the plasma membrane and deep within the cytoplasm [20]. By contrast, GV-stage human oocytes obtained from very early antral follicles in unstimulated ovaries presented CGs in the subplasmalemma and cortical cytoplasm of fully grown, immature oocytes derived from very early antral follicles [33]. The group of Gethler [34] identified dark and light subpopulations of CGs in leftover human GV-stage oocytes collected after a “long” protocol (GnRH analogue and human menopausal gonadotrophin, hMG) for ovulation induction. Many clusters of light granules were distributed throughout the cytoplasm, with a few granules (mainly dark) at the cortex. Cryopreservation by slow-freezing induced a decrease in the dark granule’s abundance, with membrane-coated electron-transparent vesicles, in some cases aggregated with CGs [34]. We, similarly, found scattered in the ooplasm CGs in both fresh and vitrified-warmed immature leftover GV-stage oocytes, with a higher counterpart of dark granules (indicative of a better maturity), probably due to the different protocol for cryopreservation [22]. In immature oocytes from PCOS patients, CGs were dispersed throughout the whole cytoplasm, without being prominent [31].

#### 3.2.5. Vacuoles

Some vacuoles with a diameter of 1–2 µm were observed in the center of the oocytes [20]. Numerous membrane-bounded vacuoles, small and empty (medium diameter ± SD: 0.975 ± 0.113 µm), were found mainly in the region surrounding the nucleus of human leftover GV-stage oocytes after full stimulation [22]. Similarly, vacuolar structures were very abundant and with a variable ultrastructure in leftover human GV oocytes after superovulation [35]. When GV oocytes were subjected to vitrification by Cryotop, vacuoles were found also in the oocyte periphery. Numerous small MV complexes were identified in the cortex and subcortex; their number decreased in vitrified-warmed oocytes [22]. A centralized vacuolated ooplasm was connected to atresia [24]. A representative micrograph from our group of vacuoles and smaller vesicles from a fully stimulated human oocyte is shown in Figure 1B.

#### 3.2.6. Lysosomes

One author reports that primary and secondary lysosomes were not present in GV oocytes [31]; by contrast, secondary and tertiary lysosomes were commonly found in GV oocytes from stimulated cycles, and bizarre images were considered a sign of atresia [24]. The lysosomes were also found in the cytoplasm of all analyzed leftover human GV from young stimulated donors [26]. They varied in size (0.2–1.5 μm) and were described as membrane-bound bodies containing aggregated vesicles with variable opacity, membrane remnants, or dark, dense patches (probably insoluble lipid droplets), residues of lysosomal degradation [26]. Lysosomes were also noticed in preovulatory human oocytes maturing *in vitro* at the metaphase I stage from stimulated patients [25]. A representative archived micrograph of lysosomes from fully stimulated human oocytes is shown in Figure 1B.

### 3.3. Oolemma

#### Microvilli, Perivitelline Space (PVS) and Zona Pellucida (ZP) Texture

Numerous fine microvilli were observed closely on the oocyte surface after enzymatic removal of the zona pellucida in GV oocytes from unstimulated patients. Twenty-eight hours of culture determined an extension from the surface of the plasma membrane into the ZP [23]. Numerous slender microvilli, 4–5 µm long, extended from the surface of the cells [20]. A continuous layer of thin and long microvilli covered the oocyte surface in leftover human GV-stage oocytes after full stimulation; by contrast, after vitrification, an irregular coverage of small and short microvilli was noted in the 40% of observed oocytes [22]. Moreover, when GV-stage oocytes were collected from a PCOS patient after COS, microvilli were rare and small [28]. In GV-stage oocytes collected from a PCOS patient, the PVS was very narrow, the outlines of the ZP were sharp and the ZP was less dense in the exterior and denser in the interior [28].

**Figure 2:**
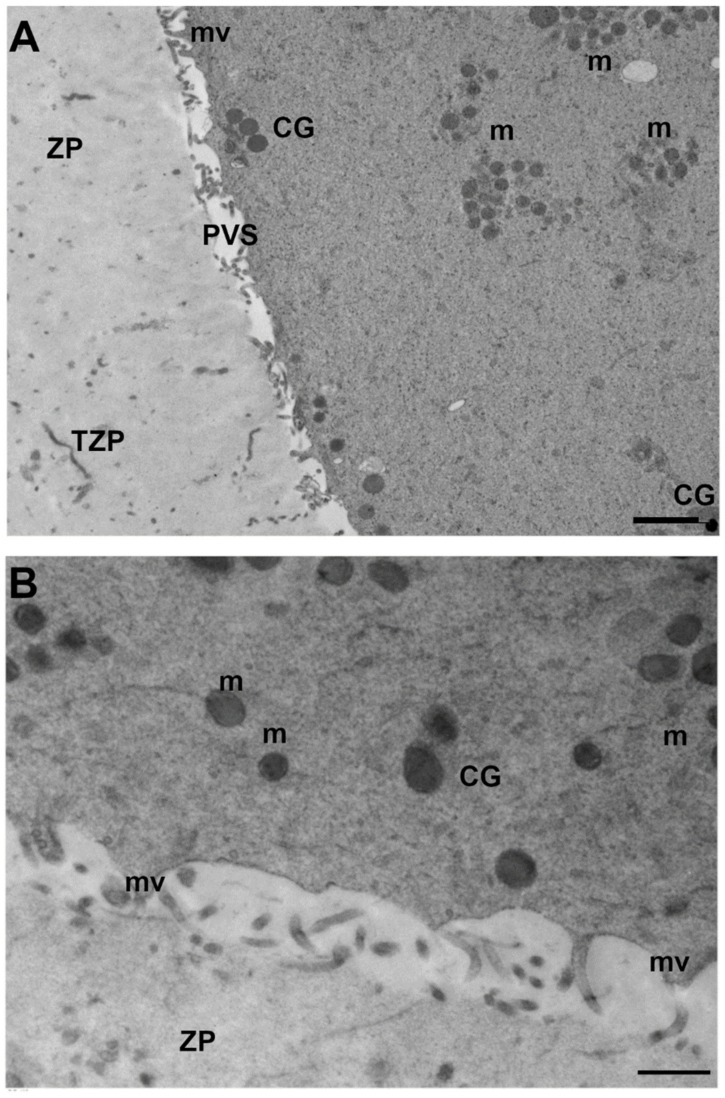
Representative unpublished archived TEM micrographs of GV-stage human oocytes from stimulated ovaries [22,29,30] showing in (**A**) the cortex with a pale and spongy zona pellucida (ZP) crossed by numerous residues of transzonal projections (TZP) and a thinner perivitelline space (PVS) in which numerous thin microvilli (mv) protruding from the oolemma are visible. The ooplasm is characterized by the rare presence of suboolemmal cortical granules (CG) and numerous mitochondria (m), which are often present in clusters in the inner ooplasm. TEM, bar: 1 µm; (**B**) higher magnification of the cortical ooplasm with round-to-ovoid mitochondria (m), slightly electron-dense cristae and rare cortical granules (CG). The oolemma is folded in numerous microvilli (mv) protruding in the perivitelline space. ZP: zona pellucida. TEM, bar: 800 nm.

Immature oocytes retrieved from an *in vitro* fertilization program showed, by SEM, a smooth and compact surface of the zona pellucida, different from the spongy appearance in mature oocytes [36]. Human GV oocytes show corona radiata processes extending through the ZP, some ending in bulbous or club-shaped terminals containing microfilaments, granular vesicles, and a few lysosomes [25]. A representative micrograph of the cortical ooplasm, oolemma, microvilli, PVS and ZP from fully stimulated human oocytes is shown in Figure 2.

## 4. Oocyte Quality and Early Embryo Development from ART Cycles

After ovulation triggering through hCG administration, the oocyte retrieval results in the collection of both mature MII-stage oocytes (~70–85%) and immature GV-/MI-stage oocytes (up to 30%) [37,38,39]. The number of immature oocytes, when high after transvaginal retrieval, is generally considered a marker of poor oocyte quality [40,41], and can increase exponentially in PCOS patients [42,43]. During the next steps of IVF, the oocyte quality covers a pivotal role in determining embryonic development and in increasing the chances of a successful clinical pregnancy. The definition of oocyte quality is dependent on its potential and ability to undergo meiotic maturation and fertilization process, to achieve the proper embryonic development and to further clinical pregnancy [44]. The quality of human GV-stage oocytes can be evaluated by morphological markers such as the surrounded nucleolus (SN) and non-SN, which identify high and low developmental potential cells and consequent IVM outcomes [45]. Literature data, although limited, provide the scientific and medical community with information about the importance of GV-oocyte quality utilized in IVF programs. Escrich and collaborators [46] identified two oocyte populations by real time-lapse recording, defined on the early or late timings of maturation (E-IVM oocytes: less than 20 h; and L-IVM oocytes: up to 30 h, respectively), which were mainly affected by the hours needed for the GVBD. While E-IVM oocytes reached the activation rate and pronucleus (PN) formation in a percentage comparable to *in vivo* matured oocytes, the L-IVM group showed abnormal patterns in chromatin segregation that impair normal PN formation and, thus, fertilization rates and embryo formation/development [46]. The importance of maturation timings was confirmed by Yang and collaborators, who additionally highlighted the role of serum progesterone levels, as well as GV-MI maturation, for a better developmental potential of IVM oocytes [47]. Furthermore, the quality of immature oocytes before and during IVM is pivotal for increasing the chances of success in women with low functional ovarian reserve. The application of a correct maturation of these oocytes can add the +60% of the total embryos available for transfer, providing an improvement in pregnancy and delivery chances [48]. On the other hand, a non-randomized prospective trial [49] suggested double ovarian stimulation (DuoStim) as an alternative solution to the rescue IVM of oocytes retrieved from ovarian reserve poor-prognosis women. The application of DuoStim resulted in higher efficiency in terms of oocyte competence, embryo viability and quality [49]. Due to the reasons here discussed, the use of GV-stage oocytes is still limited in routine clinical practice; thus, more studies are needed on this topic to provide a deeper knowledge of its potential applications in ARTs.

## 5. Unstimulated Vs. Stimulated GV-Stage Oocytes

The oocyte retrieval in natural cycles has been the main source of female germ cells since the beginning of IVF history [50,51]. This procedure consists of the transvaginal aspiration of oocytes from mature follicles and, to date, is mainly applied in PCOS patients and poor responders, or highly recommended to couples in which the woman is under the age of 40 and there is no male factor infertility. Nowadays, women undergoing ARTs are subjected to COS, which allows the maturation and ovulation of multiple follicles through the injection of exogenous gonadotropins [40]. Both unstimulated and stimulated cycles display a plethora of advantages and disadvantages for the successful retrieval of mature oocytes. Regarding natural unstimulated cycles, the advantages reside in decreasing the risk of ovarian hyperstimulation syndrome (OHSS), costs, and in avoiding the use of hormones for ovarian stimulation; moreover, IVF cycles may be frequently repeated, thus reducing the perinatal adverse outcomes and side effects of endometrial functions [52,53,54]. However, the efficiency of unstimulated cycles is counterbalanced by a lower number of available oocytes and, therefore, of embryos to be transferred, together with decreased pregnancy rates per cycle and increased cancellation rate (more than 30%) due to premature ovulation [55]. On the other hand, the advantages of using COS in IVF cycles include an increased number of oocytes available during follicular aspiration, and thus a greater number of embryos to be transferred to the uterine cavity [56]. Nevertheless, the administration of exogenous hormones, especially at high doses or in the case of repeated IVF cycles, can decrease endometrial receptivity, increase the risk of OHSS breast cancer and is associated with higher costs per cycle [57]. In both unstimulated and stimulated cycles, a high number of immature oocytes from secondary follicles are frequently retrieved and may undergo IVM [58]. This may increase pregnancy rates, although the percentages of successful full-term pregnancy and live birth rates from immature oocytes after IVM are still very low [48]. Particularly, a more numerous retrieval of immature oocytes may be correlated with the COS protocol utilized, i.e., when Follicle Stimulating Hormone (FSH), GnRH antagonist and short GnRH agonist protocols are preferred to FSH/LH, GnRH agonist and long GnRH agonist protocols, the former characterized by the administration of GnRH agonist on cycle-day 21 followed by gonadotropin from cycle-day 2 to 14 [59,60,61]. Therefore, GV-stages oocytes from both unstimulated and stimulated follicles have special importance for those patients with a history of unsuccessful treatments, representing an alternative solution for a tailored ART optimization in a patient-friendly and -specific manner. In this regard, IVM and oocyte cryopreservation may be applied, even if the potentiality of using immature oocytes is not yet assessed.

## 6. *In Vitro* Maturation (IVM)

Among the relevant ARTs, oocyte-cumulus complexes can be retrieved after the aspiration of mid-sized follicles and subsequently cultured for 24–48 h for undergoing the process of IVM. Moreover, GV-stage oocytes that did not undergo the transition to MII-stage after stimulation can be found in the pool of retrieved oocytes and subjected to IVM [62]. The IVM of these “leftover” immature (GV-stage) oocytes with developmental competence represents a potential tool for ART patients, in particular for poor responders, PCOS women, and for avoiding the side effects of OHSS [29,63,64]. While discarded in the past, today GV oocytes can be cryopreserved before or after IVM, thus increasing the opportunity for achieving a pregnancy [19,29]. However, the successful application of IVM on leftover oocytes is still facing issues, mainly due to defects in the cytoplasmic maturation or asynchronous cytoplasmic and nuclear maturation [30,46,65]. Additionally, the delayed timings of fertilization after MII arrest and meiotic spindle assembly significantly impair embryo developmental potential [30,66]. Notably, the IVM rates of GV-stage oocytes from unstimulated and stimulated cycles highlight differences that seem to be strictly associated with culture media, age, infertility factor and the presence of cumulus cells [67].

### 6.1. IVM of Unstimulated GV-Stage Oocytes

The first live birth after IVM resulted from oocytes retrieved from an unstimulated woman affected by PCOS and was reported in 1994 by Trounson and collaborators [68]. The IVM process is similar in stimulated and unstimulated cycles, although in the latter it appears to be less efficient, due to a lower number of retrieved oocytes and a decrease in both cleavage and pregnancy rates [69,70]. However, pilot studies highlighted differences between the IVM of stimulated and unstimulated oocytes, as structural and molecular changes [71], and a protein synthesis profile that leads to different germinal vesicle break-down (GVBD) time courses [72]. IVM of immature oocytes from unstimulated cycles evidenced smaller diameters after denudation (96–125 µm) and decreased *in vitro* competence than in stimulated cycles [73,74]. As a consequence, hCG priming before oocyte retrieval in control and PCO/PCOS patients increased oocyte maturation and fertilization rates, developmental potential and clinical pregnancy rates, when compared to unstimulated cycles [75,76]. Liu and collaborators scored GV- and MI-stage oocytes from unstimulated PCOS patients according to nuclear maturation, cumulus cells-oocyte shape and morphology. Maturation, fertilization and cleavage rates were similar between the two groups, while it is worth noting that the number of retrieved GV-stage oocytes showed a threefold increase in comparison with MI-stage oocytes [77]. This reinforces the need to better optimize ART settings for GV-stage oocytes.

### 6.2. Rescue IVM from Stimulated Oocytes

Nowadays, new strategies are still needed for low functional ovarian reserve patients with abnormally elevated FSH, together with abnormally low age-specific anti-mullerian hormone (AMH) levels [78], and for poor responder patients [64]. One of the clinical issues of these women is poor oocyte quantity and quality after retrieval. Approximately, up to 30% of the retrieved oocytes are immature (GV- or MI-stage) and need to be matured *in vitro* to increase the possibilities of a successful IVF cycle [79]. Oocytes that matured on Day 1 after retrieval were significantly higher in quality than those that matured later on [80]. Hormonal supplementation with key hormones as Epidermal Growth Factor (EGF) or EGF-like growth factors such as amphiregulin (AREG) and epiregulin (EREG), improved meiotic resumption and nuclear maturation [81]. Notably, a significantly higher percentage of GV-stage oocytes that matured, as evidenced by PBI extrusion, were detected when GVBD occurred in the central nucleolus rather than in the peripheral nucleolus [82]. As long as this procedure is considered a valuable tool for increasing the chances of successful fertilization and embryo development, it is worth mentioning that rescue IVM of GV oocytes could negatively influence embryo morphokinetics, with particular regard to the timings of the overall embryo formation and development and cleavage patterns [83].

## 7. Cryopreservation

One of the major debates regarding fertility preservation concerns the cryopreservation of GV-stage oocytes before or after IVM when they will reach the MII-stage. In 1998, Tucker and collaborators published the first birth from GV-stage oocytes that were first cryopreserved and, after thawing, matured *in vitro* [83]. After ICSI and the achievement of the cleavage stage, the newly formed embryos underwent embryo transfer and resulted in a live birth of a female infant [84]. The role of cumulus cells during immature oocyte cryopreservation is still unclear [85,86,87]. The presence of somatic cells during oocyte cryopreservation may exert a protective role against anisotonic conditions and stress derived from osmotic changes, mainly induced by the rapid influx and efflux of cryoprotectants during pre-freeze and post-thawing periods [88]. Among the different procedures of cryopreservation, the two methods mostly studied and applied in the clinical daily processes are the slow freezing and vitrification methods, further discussed in this section.

### 7.1. Slow Freezing

The slow-freezing method on GV-stage oocytes is an alternative strategy of cryopreservation aimed at avoiding spindle depolymerization, decreasing the risk of polyploidy and/or aneuploidies. The reason relies on the diffuse chromatin status at this stage of oocyte maturation and the presence of the nuclear membrane surrounding and protecting the DNA [89]. This method improves oocyte survival rates, and does not, concomitantly, increase the rates of maturation and fertilization or embryonic development [90,91]. The advent of vitrification, quick, economic and safe, progressively reduced the use of slow freezing, more subject to the formation of ice crystals in the intra- and extracellular spaces [92]. The warming/thawing process countersigned the main significant difference between slow freezing and vitrification, with rapid warming being more efficient in terms of survival rates than rapid thawing [93]. However, a study of GV and MII oocytes subjected to the two different cryopreservation methodologies found no difference in terms of mitochondrial distribution and viability rates [94].

### 7.2. Vitrification

Among cryopreservation protocols, vitrification is the gold standard for the oocyte and embryo “freeze-all” strategy [95]. It can be performed by using either open or closed systems, which are in direct and indirect contact with liquid nitrogen, respectively [96,97]. Regarding slow freezing, the use of vitrification achieved better oocyte survival and higher pregnancy rates [98,99]. Oocyte maturation rates were reduced in GV-stage oocytes subjected to IVM after cryopreservation in comparison with fresh *in vitro* matured oocytes [100]. Nevertheless, considering the statistics regarding healthy live births, the literature data demonstrate that vitrification of oocytes subjected to IVM is still less effective compared to its *in vivo* ovulated counterpart [29]. Due to the use of high concentrations of cryoprotectants for oocyte vitrification and the high cytotoxicity of these chemicals, linked to osmotic changes [101], the application of vitrification and the following clinical efficiency needs to be further investigated and be strengthened with multiple controlled clinical trials [102].

## 8. Mitochondrial Replacement

One of the latest ARTs is mitochondrial replacement therapy (MRT). The main function of mitochondria in eukaryotic cells is the production of ATP through oxidative phosphorylation. Being the only semi-autonomous organelle of the cell, the mitochondrion carries up to 10 copies of mitochondrial DNA (mtDNA), which represent around 0.1% of the genome present in a cell [103]. The mtDNA mutations, which can be inherited only from the maternal counterpart, may cause severely debilitating syndromes in newborns, or lead to abortion during pregnancy [104,105]. Besides the clinical difficulties in the MRT, it faces theological and ethical issues because of the involvement of a mitochondrial donor in embryo formation [106]. Thus, the newborn will have three distinct genetic materials: the paternal through the spermatozoon, the maternal through the nuclear DNA of the oocyte, and the donor through the healthy cytoplasmic mtDNA, ultimately generating a “three-parent baby” [107]. To date, several techniques are eligible for MRT, which consists preferably of the removal of the nuclear genome from the oocyte or zygote carrying the mtDNA mutation and transferring to a healthy enucleated oocyte [108,109]. Among the MRT techniques, ooplasm transfer (OT), spindle transfer (ST), pronuclear transfer (PNT) and polar body transfer (PBT) are the most widely known and used in ART procedures [107,110]. The OT technique consists of the transfer of 5–15% of the ooplasm of a healthy donated oocyte to the oocyte-carrying mutated mtDNA and is one of the most controversial among MRTs [111]. However, OT treatment resulted in 50 clinical pregnancies and more than 30 live births [109,112]. The transfer of the pathological maternal MII-stage oocytes spindle to a healthy donor’s ooplasm previously deprived of its spindle has been applied to human oocytes by Tachibana and collaborators in 2013 [113]. The first clinical pregnancy leading to a live birth using ST was reported in 2017 [114]. Interestingly, germinal vesicle transfer (GVT) is also included among the MRT techniques, even if to date only two studies on mouse models have been reported, with unpromising results [115,116]. Briefly, GVT consists of the transplantation of the GV from a patient’s immature oocyte to a healthy donor enucleated one. The pivotal issue of this technique resides in the need of subjecting the new immature oocytes to IVM for obtaining a mature oocyte ready for fertilization.

## 9. Conclusions

ART laboratories might improve the use of immature GV-stage oocytes for further applications during routine procedures due to an increasing percentage of women unable to achieve oocyte maturation in unstimulated or stimulated cycles. The current application of GV-stage oocytes in ARTs is still limited and a matter of debate, underlining the necessity of morphofunctional studies for shedding light on the correct use of these “leftover” oocytes. TEM and SEM are the most powerful tools for assessing the fine structure of immature human oocytes, allowing the qualitative evaluation of pivotal organelles such as the nucleus, mitochondria, vacuoles and lysosomes before or after COS protocols, IVM and freezing strategies. However, due to the limited number of available leftover oocytes for these purposes, more studies are necessary for clarifying the most efficient use of GV-stage oocytes for ART optimization.

## Data Availability

The data that support the findings of this study are available from the corresponding author upon reasonable request.
